# Evaluation of groundwater quality in the rural environment using geostatistical analysis and WebGIS methods in a Hungarian settlement, Báránd

**DOI:** 10.1007/s11356-023-28627-1

**Published:** 2023-07-13

**Authors:** Dániel Balla, Emőke Kiss, Marianna Zichar, Tamás Mester

**Affiliations:** 1 Department of Data Science and Visualization, Faculty of Informatics, University of Debrecen, Debrecen, H-4028 Hungary; 2 Department of Landscape Protection and Environmental Geography, University of Debrecen, Debrecen, H-4032 Hungary

**Keywords:** WebGIS, Cleaning process, Water quality index, Degree of contamination, Cloud, Hungary

## Abstract

The evaluation, visualization of environmental data from long-term monitoring, and making them accessible in a processed form in user-friendly interfaces on the Internet are important tasks of our time. The pollution of groundwater resources in settlements is a global phenomenon, the mitigation of which requires a number of environmental measures. In this study, water quality changes following the construction of a sewerage network were examined in the course of long-term monitoring between 2013 and 2022, during which 40 municipal groundwater wells were regularly sampled. Classifying the monitoring data into pollution categories based on water quality index (WQI) and degree of contamination index (Cd), a high degree of contamination was found in the period before the installation of the sewerage network (2014), as the majority of the wells were classified as contaminated and heavily contaminated. In the monitoring period following the installation of the sewerage network, a significant positive change was found in the case of most of the water chemical parameters tested (EC, NH_4_^+^, NO_2_^−^, NO_3_^−^, PO_4_^3−^). Based on interpolated maps, it was found that an increasing part of the area shows satisfactory or good water quality. This was confirmed by the discriminant analysis as well, as it is possible to determine with an accuracy of 80.4% whether the given sample originates from the period before or after the installation of the sewerage network based on the given water chemical parameters. However, 8 years after setting up the sewerage network, the concentration of inorganic nitrogen forms and organic matter remains high, indicating that the accumulated pollutants in the area are still present. To understand the dynamics of purification processes, additional, long-term monitoring is necessary. Making these data available to members of the society can contribute to appropriate environmental measures and strategies.

## Introduction

The mapping, monitoring, and elimination of the underground sources of pollution in settlements has become a key environmental task of the twenty-first century in both developing and developed countries (Bano et al. 2022b). The importance of the topic is indicated by the fact that the demand for fresh water has increased on a global scale over the past two decades, thanks to population growth, urbanization and intensive industrial and agricultural activities (Kerényi and McIntosh 2020). International studies show that one of the most significant sources of pollution in settlements is municipal wastewater, the treatment of which is often unresolved due to a lack of financial resources (Jumma et al. 2012; Ravikumar and Somashekar 2012; Machiwal and Jha 2015; Richards et al. 2016; Smoroń 2016; Adimalla and Qian 2021; Rather et al. 2023). Several studies find that due to the lack of wastewater treatment systems in rural areas, wastewater is discharged into groundwater, as a result of which groundwater quality is severely degraded (Backman et al. 1998; Rotaru and Răileanu 2008; Nemčić-Jurec et al. 2019). Moreover, the accumulation of pollutants in soils exacerbates water contamination, as it can provide a long-term supply of pollutants (Rather et al. 2022; Bano et al. 2022a). In addition, the increase in the permanent population of settlements, in parallel with the global climatic change have led to the decline in water resources, increasing the vulnerability of groundwater (Nlend et al. 2018; Abdalla and Khalil 2018). The water quality studies following the construction of the sewerage network confirmed the positive changes in groundwater pollution in addition to our previous research, mainly in case studies conducted in urban environments (Fylypchuk et al. 2017; Mester et al. 2017; Bugajski et al. 2019; Schuler et al. 2019; Ahmad and Ghanem 2021).

The scientific basis for the combined qualitative assessment of chemical, physical, and biological parameters describing water quality was first made possible by the water quality index introduced by Horton (Horton 1965). The most important goal of the practical use of indices is to combine the applied water chemical and water physical parameters into a single number that interprets information about the state of water quality (Ball and Church 1980; Bouslah et al. 2017). The water quality index (WQI), developed by Brown et al. (1970), which calculates input parameters by weighted averaging, has been modified or improved several times over the past fifty years by specialists (Brown et al. 1970). Other indices have been developed based on WQI, e.g., US National Sanitation Foundation Water Quality Index (NSFWQI), Oregon and British Columbia indices (OWQI, BCWQI), Smith’s index, overall index of pollution (OIP), The River Ganga Index, recreational water quality index (RWQI), contamination index (Cd), and Dinius Water Quality Index (DWQI) (Prati et al. 1971; Ott 1978; Dunnette 1979; Dinius 1987; Smith 1987, 1990; Sharifi 1990; Backman et al. 1998; CPCB 2000; Cude 2001; Lumb et al. 2006; Swamee and Tyagi 2007; Kannel et al. 2007; Salim et al. 2009). The obtained water quality data, integrated and visualized into modern GIS-based decision support systems, further assist in the designation of new monitoring wells, the design and expansion of the monitoring network, and the elimination of detected pollution (Güler et al. 2002; Majolagbe et al. 2016).

The WMS-based (Web Map Service) data provision of international and national geodatabases related to water quality is of great importance for education, research, and industry. In line with current technological trends, typically cloud-based Web monitoring/forecasting and decision support systems provide access to spatial and temporal data of these databases, and the possibility of analyzing them in real time (Evangelidis et al. 2014; La Guardia et al. 2021; Pasquaré Mariotto et al. 2021; Balla et al. 2022). As a result, the publication of spatial data on the Web has shifted to infocommunication interfaces, where the easy-to-use and interpretable “map-based” query interface is a primary consideration (Jiang and Li 2005; Farkas 2017). The rise of digitalization (referred to as “geoinformatic revolution”) has induced a technological generational change (Balla et al. 2022). Static solutions are replaced now by cloud-based solutions for WebGIS architectures to support the free availability of the results of analysis of robust spatial databases (Schütze and Vatterrott 2007; Pődör and Zentai 2017; Farkas 2017; Rumiński et al. 2019; Pőddör and Szabó 2021). The concept of geovisualization of spatiotemporal data has been present in cartography literature since the 1950s (Philbrick 1953). Nevertheless, it gained more attention in the 1980s when scientists from various disciplines began exploring the possible uses of scientific visualization (Taylor 1991; Maceachren and Kraak 1997; Arabnia 1999). According to McCormick et al., geovisualization aims to visually represent the data collected by researchers, aiding in the interpretation of the information encoded within the data (McCormick et al. 1987). Scientific visualization is not limited to spatiotemporal data and is utilized in various other fields (MacEachren et al. 2004; Munzner 2008). Concepts to scientific visualization include information visualization and geovisualization, which focuses on spatial data. Information visualization primarily revolves around interactive demonstrations to aid human cognition. In contrast, geovisualization involves developing visualization methods for spatial data using GIS techniques and tools. The term geovisualization was coined by MacEachren by contracting the expression “geographic visualization” and involves a new approach to the use of maps. Maps are not created for public use but rather for individual use, primarily to provide new insights from the data. Geovisualization involves an intensive interaction between people and maps, allowing for direct manipulation of spatial data to be mapped. Maps are not used alone but are often combined with other visual aids (MacEachren et al. 2004). As geovisualization accelerated, the ability to navigate in different software environments and on Web maps became also important in the research society, which was gradually included among digital competencies, confirmed by numerous inter- and multidisciplinary research projects (Balla et al. 2022; Yang et al. 2022; Papadopoulou et al. 2022).

Publications have already been published on contamination and the deterioration of water quality caused by wastewater in urban environments; however, comparative water quality studies based on long-term monitoring data have yet to be carried out. To assess changes in the water quality status of a municipality over space and time, knowledge of the reference status is required, although this is not available in most cases. In addition, the lack of spatial and temporal monitoring data covering the settlement and the lack of geodatabases of water quality with local dimensions further complicates the study of the dynamics of the purification processes. For this reason, the primary goal of our research is to present the results of a research related to the above problem. The novelty of our study lies, among other things, in the fact that although the multidisciplinary application of various WebGIS services enjoys great popularity, the complex geospatial processing and cloud-based web geovisualization of water quality data have not been in the focus of research so far. Based on the above, the most important goal aims of the research is to determine whether the quality of groundwater has significantly improved in the settlement in the period following the construction of the sewerage network, with the help of freely available Web-based geovisualization tools and geostatistical data analysis.

## Material and methods

### Description of study area

**Fig. 1:**
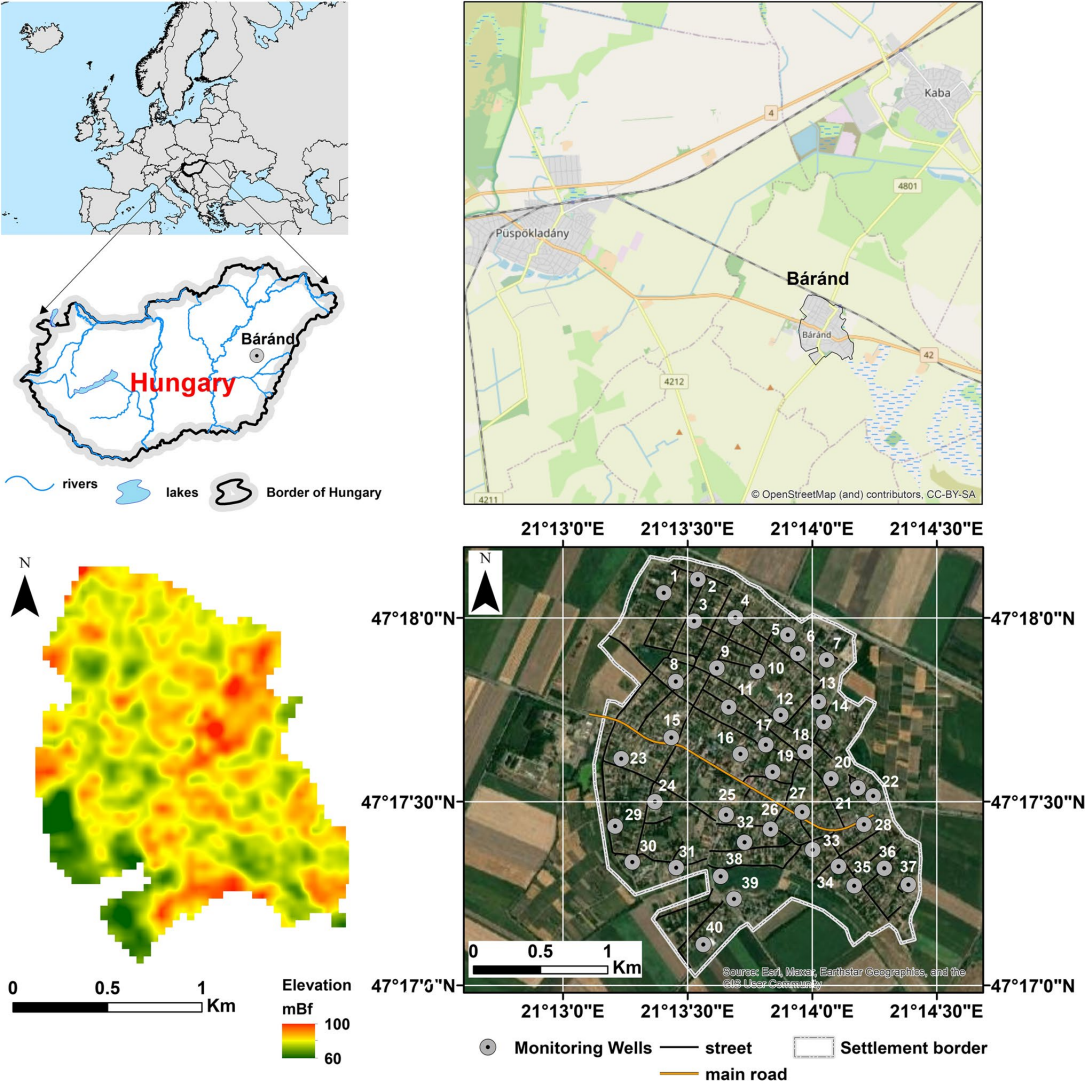
Location of study area with 40 monitoring wells

The Eastern European settlement included in the study is located in Nagy-Sárrét in the eastern part of the Great Hungarian Plain, Hungary (Fig. 1). Báránd is a typical middle-sized village, with a permanent population of 2491 people (Hungarian Central Statistical Office). As the discharge of sewage in the municipality exceeds 2000 population equivalent (PE), it belongs to the group of municipalities for which the European Union requires the construction of a sewerage network (2000/60/EC 2000). The sewerage network was completed in 2014 with the support of the Environment and Energy Operative Program of the New Hungary Development Plan. By 2022, 8 years after the construction of the sewerage network, more than 90% of households had connected to the grid, but there are still several households that have not complied with the law. Previous significant pollutant emissions have led to a strong contamination of groundwater resources and a significant deterioration in water quality, which is monitored by regular annual sampling to identify potentially contaminated sites and evaluate purification processes (Mester et al. 2017, 2019, 2020; Balla et al. 2022).

### Determination and evaluation of water quality indices

In our research 40 groundwater monitoring wells were investigated in the study area. Water samples were collected from the upper 1 m water column of each well. In order to calculated the water quality indices, we were used eight parameters (pH, EC, NH_4_^+^, NO_2_^−^, NO_3_^−^, PO_4_^3−^, COD, Na^+^). Water samples were determined using appropriate procedures according to the Hungarian Standards (HS ISO 7150-1 1992; HS 448-18 2009; HS 1484-13 2009).

#### WQI

Calculation of the WQI was carried out following the “weighted arithmetic index method” using the equation (Brown et al. 1970):

$$WQI=\sum {Q}_n{W}_n/\sum {W}_n$$

where *Q*_*n*_ is the quality rating of the *n*th water quality parameter, and *W*_*n*_ is the unit weight of the nth water quality parameter. The quality rating *Q*_*n*_ is calculated using the equation:

$${Q}_n=100\left[\left({V}_n-{V}_i\right)/\left({V}_s-{V}_i\right)\right]$$

where *V*_*n*_ is the actual amount of the *n*th parameter present, *V*_*i*_ is the ideal value of the parameter [V_i_ = 0, except for pH (V_i_ = 7)], and ***V***_***s***_ is the standard permissible value for the nth water quality parameter. The unit weight (*W*_*n*_) is calculated using the formula:

$${W}_n=k/{V}_s$$

where *k* is the constant of proportionality and is calculated using the equation:

$$k=\left[1/\sum 1/{V}_s=1,2,\dots, n\right]$$

#### Cd

The calculation of the contamination degree, Cd, is made separately for each sample of water analyzed, as a sum of the contamination factors of individual components exceeding the upper permissible value. Hence, the contamination index summarizes the combined effects of several quality parameters considered harmful to household water (Rapant et al. 1995).

The scheme for the calculation of Cd is as follows:

$${\textrm{C}}_{\textrm{d}}=\sum\limits_{\textrm{i}=1}^{\textrm{n}}\ {\textrm{C}}_{\textrm{fi}}$$

where

$${\textrm{C}}_{\textrm{fi}}=\frac{{\textrm{C}}_{\textrm{Ai}}}{{\textrm{C}}_{\textrm{Ni}}}-1$$

C_fi_: contamination factor for the *i*th component
C_Ai_: analytical value of the *i*th component
C_Ni_: upper permissible concentration of the *i*th component (N denotes the “normative” value)

The elements and ionic species with analytical values below the upper permissible concentration values are not taken into consideration.

The evaluation of the indices is shown in Table 1.

**Table 1:** WQI range, WQS status, Cd range, Cd status and possible use of the water sample

Rank	Water quality index (WQI)	Water quality status (WQS)	Contamination degree	Contamination degree status (Cds)
Rank 1	0–25	Excellent	0	Excellent
Rank 2	26–50	Good	< 1	Low
Rank 3	51–75	Poor	3–1	Medium
Rank 4	76–100	Very poor	6–3	High
Rank 5	Above 100	Unsuitable for any use	> 6	Very high

### Geoprocessing and web sharing

**Fig. 2:**
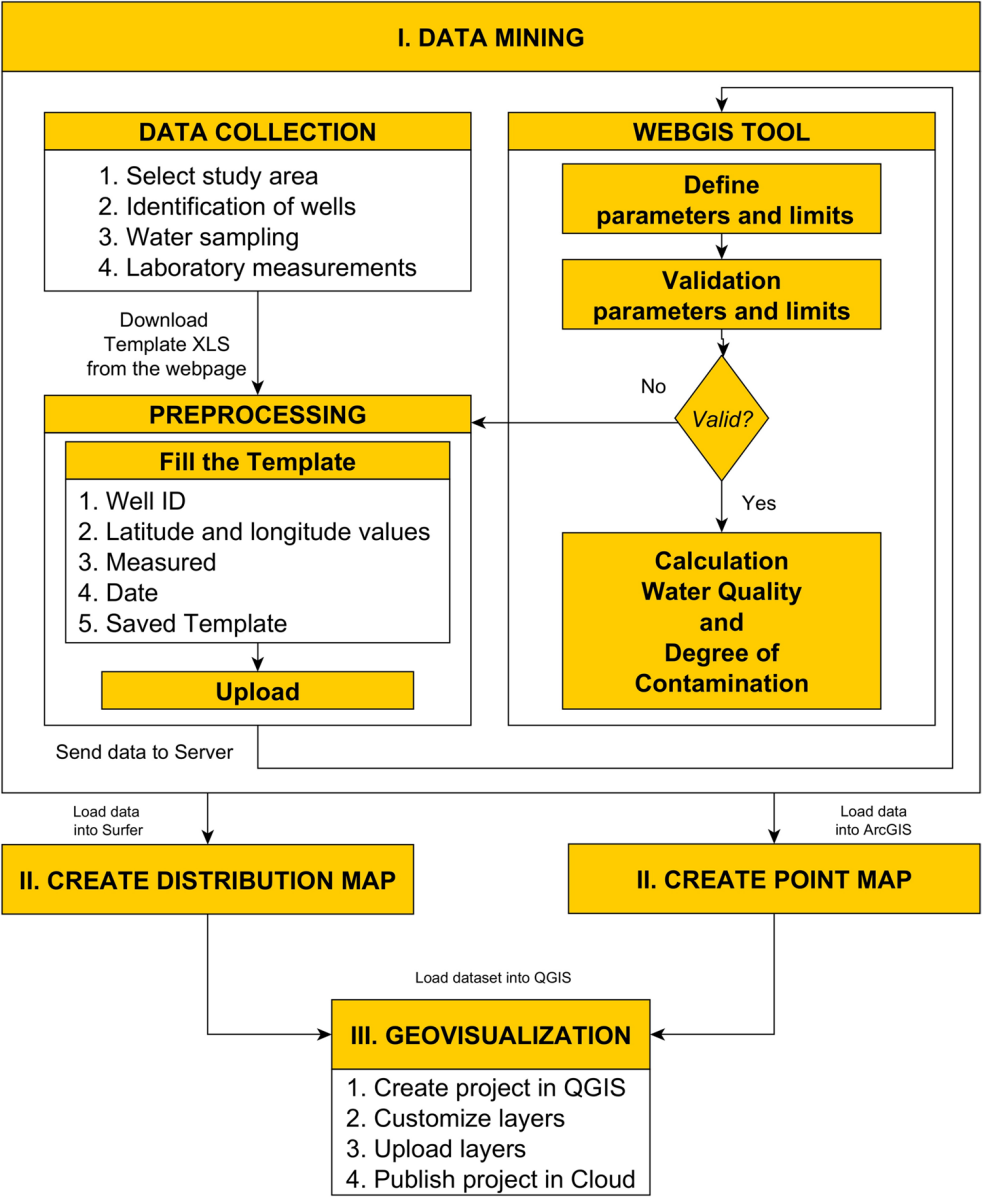
Geoprocessing and Web-sharing

The mapping of the water quality status of the settlement and sharing the results on a cloud-based WebGIS platform was realized as a result of three work phases ((I) data collection and forming the database, (II) map editing, and (III) Web geovisualization) (Fig. 2). During the data collection, laboratory measurements of the taken water samples and the determination of the water quality indices (water quality index, contamination degree) were carried out using a proprietary webGIS tool (Balla et al. 2022). Geospatial processing of time-series water quality data exported from the system was performed using ESRI ArcGIS version 10.4.1 (ESRI 2011). Since water chemistry and water quality data from the wells were available in a table along with coordinates, it was necessary to import them into the Geographic Information System (GIS). From the data of the water quality status, distribution maps were made of the area for each time point using kriging interpolation, which were saved as raster files in GeoTiff format using Surfer version 22 software (Surfer® from Golden Software LLC, n.d.). The web sharing of the created raster and vector layers in a QGIS environment was geovisualized and made freely available using the QGIS Cloud module (QGIS n.d.).

### Statistical analysis

When developing the time-series water quality database, the measurement and calculation (water quality index values, water quality statuses) results were grouped according to the years, pre-sewerage and post-sewerage periods. In addition to calculating the basic statistical values (mean, lower, upper quartile, mode, median, and standard deviation), the results were plotted on scatterplot, boxplot diagrams for efficient data visualization.

To determine the strength of the relationship between the variables, cross-correlation studies were performed. In the non-permanent Spearman rank correlation study, the correlation was determined by the difference in the rank numbers of the variables (Spearman 1961).

In order to spatially distinguish the state of the monitoring wells of the settlement before and after the construction of the sewerage network discriminant analysis was performed. The examination was performed using Wilks' Lambda method (Nath and Pavur 1985). Fisher’s two-group discriminant analysis is suitable for separating different groups. Based on the discriminant function, the variable that defined the separation the most can be given as well (Fisher 1936).

The differences in the data sets for different dates were investigated using a Wilcoxon test. The test can be used to determine whether the differences in the data sets are random or created by some background process (Wilcoxon 1992).

Statistical processing of the data, as well as visualization of the results, was carried out using IBM SPSS 22 software. The statistical examinations are summarized in Table 2.

**Table 2:** Applied statistical methods and their aims

Statistical analysis	Aim of application	Input data
Spearman’s rank correlation	Determination of the strength of the relationship between the indices.	WQI index value, Cd index value
Wilcoxon paired test	Whether differences between the datasets are random or influenced by some kind of a background process.	Water quality status (WQS), Contamination degree status (Cds)
Discriminant analysis	Studying differentiation in space and time based on parameters.	WQI index value, Cd index value

## Results

### Description of investigated parameters

The connection of households to the sewerage network has been carried out continuously in the studied settlement since 2014. The impact of the installation of the sewerage network on water quality was the focus of our research. The reference conditions were determined with the sampling of 40 groundwater wells in the summer of 2013, the year before the construction of the sewerage network. Subsequently, sampling was repeated in the 3rd, 4th, 5th, 7th, and 8th years following the installation of the sewerage network out at the same time of the year, in the summer. Sampling in year 6 (2020) was not possible due to the COVID-19 pandemic. Based on the measurement data of 8 soil chemical parameters (pH, EC, NH_4_^+^, NO_2_^−^, NO_3_^−^, PO_4_^3−^, COD, Na^+^) measured in the samples taken in the year before (2013) and in the years after (2017, 2018, 2019, 2021, 2022) the construction of the sewerage network, the minimum, maximum, mean, as well as the lower and upper quartile values (Table 3) of the data were determined.

**Table 3:** Descriptive statistics of the studied water chemical parameters

	pH	EC (μS/cm)	NH_4_^+^ (mg/L)	NO_2_^−^ (mg/L)	NO_3_^−^ (mg/L)	PO_4_^3−^ (mg/L)	COD (mg/L)	Na^+^ (mg/L)
2013								
*N*	40	40	40	40	40	40	40	40
Mean	8.25	3032.65	0.69	0.31	187.83	1.22	6.85	237.91
Minimum	7.23	340	0.23	0.02	2.36	0.07	2.40	8.90
Maximum	9.42	7670	1.89	1.28	564.82	4.07	18.20	653.20
Lower *Q*	7.92	1950.25	0.43	0.05	50.16	0.37	4.03	132.18
Upper *Q*	8.56	4310	0.87	0.42	341.77	1.75	8.05	312.78
Limit	6.5–8.5	2500	0.5	0.5	50	0.5	4.5	200
2017								
*N*	40	40	40	40	40	40	40	40
Mean	7.51	2845.78	0.53	0.20	142.65	0.39	7.65	377.94
Minimum	7.02	876	0.08	0.01	4.46	0.03	2.90	75.80
Maximum	8.30	9290	3.42	1.86	616.64	1.54	17.68	2254.20
Lower *Q*	7.29	1871.25	0.24	0.02	37.65	0.09	5.24	154.15
Upper *Q*	7.74	3507.5	0.64	0.17	221.08	0.58	9.90	422.48
Limit	6.5–8.5	2500	0.5	0.5	50	0.5	4.5	200
2018								
*N*	40	40	40	40	40	40	40	40
Mean	8.00	2637.8	0.65	0.21	109.76	0.65	7.16	352.37
Minimum	7.21	1140	0.14	0	6.95	0.03	1.10	97.39
Maximum	8.87	6380	3.97	1.37	538.30	2.76	36.80	1828.03
Lower *Q*	7.68	1874.25	0.33	0.01	23.73	0.22	3.59	182.32
Upper *Q*	8.29	3190	0.68	0.30	153.03	0.86	8.48	447.77
Limit	6.5–8.5	2500	0.5	0.5	50	0.5	4.5	200
2019								
*N*	40	40	40	40	40	40	40	40
Mean	7.21	2773.43	0.52	0.26	170.73	0.48	7.68	383.68
Minimum	6.81	695	0.12	0	7.61	0.04	1.66	52.14
Maximum	7.90	8910	3.37	1.94	645.5	2.14	16.65	2019.77
Lower *Q*	6.97	1511.25	0.29	0.02	43.1	0.14	4.58	185.37
Upper *Q*	7.42	3792.50	0.62	0.31	244.85	0.60	10.12	477.99
Limit	6.5–8.5	2500	0.5	0.5	50	0.5	4.5	200
2021								
*N*	37	37	37	37	37	37	37	37
Mean	7.23	4128.92	0.47	0.24	164.52	0.37	4.21	319.88
Minimum	6.84	980	0.12	0.01	16.59	0.04	0.97	80.53
Maximum	8.00	14830	1.40	1.54	829.18	1.53	23.18	1396.35
Lower *Q*	7.06	1808	0.25	0.03	33.19	0.09	2.1	157.42
Upper *Q*	7.36	5930	0.51	0.31	185	0.48	4.14	362.57
Limit	6.5–8.5	2500	0.5	0.5	50	0.5	4.5	200
2022								
*N*	34	34	34	34	34	34	34	34
Mean	7.27	3664.21	0.64	0.20	296.1	0.24	2.19	153.51
Minimum	6.95	1396	0.14	0.00	9.64	0.03	0.99	21.71
Maximum	7.80	11700	2.47	1.24	1186.05	0.89	5.11	295.77
Lower *Q*	7.08	2163.75	0.38	0.04	76.1	0.1	1.71	94.90
Upper *Q*	7.50	4623.75	0.77	0.25	377.27	0.32	2.58	205.26
Limit	6.5–8.5	2500	0.5	0.5	50	0.5	4.5	200

The pH values in the pre-sewerage period were in a more alkaline range than in the post-sewerage period. The lower quartile had a value of 7.92 while the upper quartile was 8.56. In the post-sewerage years, a significant decrease in pH was detected, which can be explained by a significant decrease in anions. The average of 8.25 in 2013 fell to 7.51 in 2017, 7.26 in 2019 and 7.27 in 2021. The lower quartile of the pH value of the samples was 7.08 in 2022. The difference between the upper quartiles (2013: 8.56 → 2022: 7.5) was almost 1.

The mean values of the concentration of PO_4_^3−^ show the most significant decrease proportionally. The mean concentrations have been reduced from 1.22 mg/L in 2013 to 0.24 mg/L in 2022, decreasing therefore below the limit of 0.5 mg/L set out in the 6/2009 (IV.14.) KvVM-EüM-FVM fell joint decree (6/2009 (IV. 14.) KvVM-EüM-FVM 2009). In 2019, 32.5%, while in 2022, 11.76% of the wells had concentrations above the limit value, compared to 67.5% in 2013. The maximum value of 4.07 mg/L measured in 2013 was reduced to 2.14 mg/L in 2019 and to 0.89 mg/L in 2022.

The value of electrical conductivity after sewerage showed a significant decrease after the construction of the sewerage network until 2018, increasing again from 2019; however, the average value in 2019 (2773.43 μS/cm) was still lower than the average value for the reference year (3032.65 μS/cm). The upper quartile decreased from the pre-sewerage 4310 to 3792 μS/cm in 2019 before rising to 4623.75 by 2022. In 2022, the conductivity value in 64.71% of the wells exceeded the contamination limit of 2500 μS/cm prescribed by the 6/2009 (IV. 14.) KvVM-EüM-FVM joint decree, while it exceeded in 50% of the wells in the pre-sewerage period (6/2009 (IV. 14.) KvVM-EüM-FVM 2009). The decreasing values can be explained by the decrease of wastewater outflow, but soil conditions also contribute to the development of the total ion content and to the high values. This is also supported by an increase in Na^+^ concentrations in the post-sewerage years.

The average values of Na^+^ post-sewerage were more than 100 mg/L higher than in the reference year. For the years 2013 and 2022, both the lower quartile (132.18 mg/L → 157.42 mg/L) and the upper quartile (312.78 mg/L → 362.57 mg/L) increased. Presumably the low volumes of monthly rainfall in 2019, 2021, and 2022 could be the cause of the increase.

The amount of organic matter did not show a decreasing trend either, the average values in the post-sewerage years (2017, 2018, 2019) were usually higher than the 6.85 mg/L value in the reference year. The highest average value (7.68 mg/L) was measured in 2019. The high organic matter content measured in the 6th post-sewerage year is further evidence that a significant amount of organic matter has accumulated in the soil in the area, which continues to provide a supply of inorganic pollutants in addition to the organic matter content of groundwater. However, in the 7th and 8th post-sewerage years, the average values of the organic matter content decreased significantly (4.21 mg/L, 2.19 mg/L), which can be explained by the stopped wastewater outflow.

The mean values of NH_4_^+^ concentrations were above the limit value except for 2021, but there is a steadily decreasing trend. While in 2013 the average value was 0.69 mg/L, in 2022, the average value was 0.64 mg/L. In 2013, very high values above 1 mg/L were measured in 17.5% of the wells; this decreased to 8.82% by 2022, however, concentrations in 52.94% of wells were still above 0.5 mg/L. The strong contamination of the groundwater resources of the settlement is also indicated by the fact that concentrations below 0.2 mg/L were measured in only 2 wells.

NO_2_^−^ typically does not accumulate in waters; it is further oxidized into nitrate if a sufficient amount of dissolved oxygen is present. While concentrations above 0.5 mg/L were detected in 22.5% of the studied wells in 2013, such concentrations were found in 11.76% of the wells in 2022. The value of the upper quartile was reduced from 0.42 to 0.25 mg/L, while the value of the lower quartile was reduced from 0.05 to 0.04 mg/L. Based on this, oxidative conditions improved, which is due, among other things, to the stopped wastewater outflow. However, it should also be noted here that the concentration of nitrite is still above 0.2 mg/L in 35.29% of the wells, which indicates contamination.

The decrease in the concentration of NO_3_^−^ also indicates the elimination of a significant part of the pollutant supply, as the mean value decreased from 187.8 to 109.76 mg/L in 2018, but from 2019 onwards there was an increase in the mean value of concentrations (170.73 mg/L, 164.52 mg/L, 296.4 mg/L). The value of the lower quartile decreased from 50.16 mg/L in 2013 to 43.1 mg/L in 2019 and then increased to 76.1 mg/L in 2022, while the upper quartile decreased from 341.7 to 244.85 mg/L in 2019 and then increased to 377.27 in 2022.

### Evaluation of spatial and temporal distribution of groundwater quality and degree of contamination

For sampling times, water samples were ranked based on their quality on a scale of 1 to 5, where 1 represents the best and 5 represents the worst water rating and degree of contamination. The results of the rating are presented in Table 4. The water level in 3 and 6 monitoring wells fell below the depth of the well (4–6 m deep) in 2021 and 2022, respectively; therefore, there was a lack of data in these cases. The authors plan to establish deeper monitoring wells in the near future to maintain long-term monitoring.

**Table 4:** Categories of water samples based on water quality indices

Water quality status (WQS)						
Date	N_WQS_	Unsuitable for any usage	Very poor water quality	Poor water quality	Good water quality	Excellent water quality
2013	40	28	8	4	0	0
2017	40	9	5	14	11	1
2018	40	11	13	10	6	0
2019	40	13	6	8	12	1
2021	37	8	7	6	14	2
2022	34	6	3	14	10	1
Contamination degree status (Cds)						
Date	N_Cds_	Very high	High	Medium	Low	Noncontaminated
2013	40	18	10	9	3	0
2017	40	11	9	13	6	1
2018	40	10	11	12	6	1
2019	40	13	8	14	4	1
2021	37	9	7	12	6	3
2022	34	12	5	10	7	0

In the case of pre-sewerage (2013) sampling, no water samples were classified in rank 1 (“Excellent,” “Non-contaminated”), 3 samples were classified in rank 2 (“Low”) based on the Cd index, while no water samples were classified in this rank based on the WQI index. The strong contamination of the water of the monitoring wells is well demonstrated by the fact that 90% (N_WQS___2013___Rank4+Rank5_= 36) and 70% (N_Cds___2013___Rank4+Rank5_= 28) of the monitoring wells based on the WQI index and Cd index, respectively, were classified in rank 4 and rank 5 (“Unsuitable for any usage, Very poor–high, Very high”).

3 years after the construction of the sewerage network (2017), significant changes in water quality can be observed. For both indices, the number of monitoring wells in rank 5 indicating the most polluted samples (N_WQS___2013___Rank5_ = 28 >N_WQS___2017___Rank5_ = 9 and N_Cds___2013___Rank5_ = 18 > N_Cds___2017___Rank5_ = 11) has decreased significantly, while the number of samples in rank 2 (“Good and Low”) and rank 3 (“Poor, Medium”) has increased (N_WQS___2013___Rank2_ = 0 < N_WQS___2017___Rank2_ = 11, N_WQS___2013___Rank3_= 4 < N_WQS___2017___Rank3_ = 14 and N_Cds___2013___Rank2_ = 3 < N_Cds___2017___Rank2_ = 6, N_Cds___2013___Rank3_ = 9 < N_Cds___2017___Rank3_ = 13). In the case of the WQI index, the number of monitoring wells in the two categories increased from 4 to 25, and from 12 to 19 in the case of Cd index compared to the reference year, in addition, 1-1 monitoring wells were categorized in the best rank 1 category.

Four years after the construction of the sewerage network (2018), no significant change was detected based on the Cd index. In the case of the WQI index, the number of monitoring wells in rank 2 decreased significantly (N_WQS___2017___Rank2_ = 11 > N_WQS___2018___Rank2_ = 6), while in rank 4, an increase (N_WQS___2017___Rank4_ = 5 < N_WQS___2018___Rank4_ = 13) was found.

Five years after the construction of the sewerage network, the most significant change occurred in 2019 compared to 2018, as the number of monitoring wells in rank 4 decreased in the case of both indices (N_WQS___2018___Rank4_ =13 > N_WQS___2019___Rank4_ = 6 and N_Cds___2018___Rank4_ = 11 > N_Cds___2019___Rank4_ = 8), while the number of monitoring wells in rank 5 increased (N_WQS___2018___Rank5_ = 11 < N_WQS___2019___Rank5_ = 13 and N_Cds___2018___Rank5_ = 10 < N_Cds___2019___Rank5_ = 13). However, their number remained lower than in the pre-sewerage time in 2013. The proportion of wells in category 2-3 was 35% for the WQI index and 55% for the Cd index, compared to 30–47.5% in 2013.

Sampling was not possible 6 years after the construction of the sewerage network (2020) due to the COVID-19 pandemic.

In 2021, compared to the post-sewerage transition period (2017-2018-2019), the number of water samples in rank 1 and rank 2 increased. Positive changes in the water quality of the monitoring wells are well demonstrated by the fact that the samples of 43.24% of the monitoring wells (N_WQS___2021___Rank1+Rank2_ = 16) were classified in the categories rank 1 and rank 2 according to the WQI index. Based on the Cd index, no significant positive change was found. In addition, the number of wells in rank 5 indicating the most polluted samples decreased in the case of both indices (N_WQS___2021___Rank5_ = 8 and N_Cds___2021___Rank5_ = 9).

Based on the results of the sampling in the 8th post-sewerage year, the water quality deteriorated slightly compared to the previous year. Only one water sample was classified in rank 1 (“Excellent”) based on WQI. In addition, the number of wells in the categories “Good” or “Low” decreased compared to 2021, but their number (N_WQS___2022___Rank2_ = 10 and N_Cds___2022___Rank2_ = 7) remains higher than in the year before the construction of the sewerage network in 2013.

**Fig. 3:**
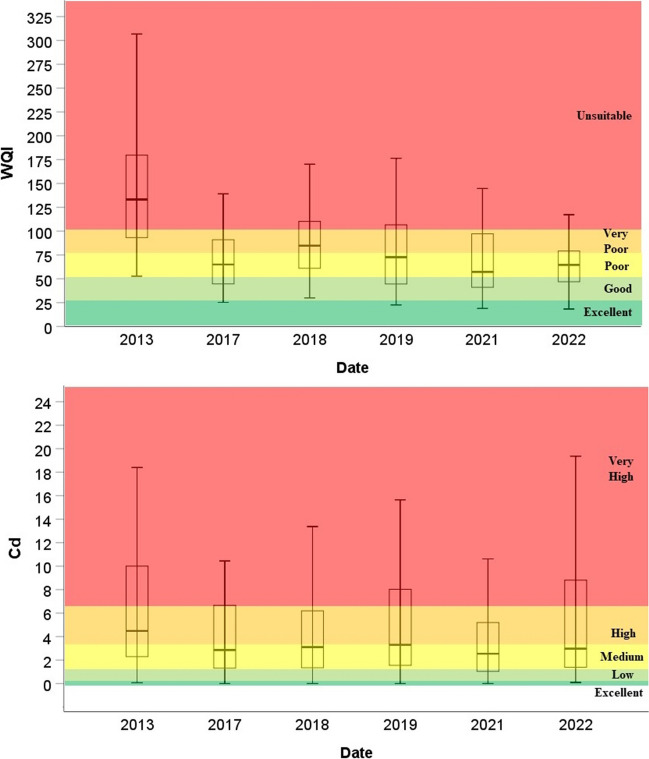
Water quality index values in the investigated periods

In order to give a detailed picture of the state of water quality and the degree of contamination, the index values determined during the water classification of each well were visualized on boxplot diagrams. For the WQS and Cd indices, lower values indicate improved water quality. Changes in the average, minimum and maximum values of both indices indicate positive changes in water quality (Fig. 3, Table 5). For WQI, the pre-sewerage average of 147.31 fell to 72.23 by 2022, and for the Cd index from 6.41 to 6.11 (Table 5).

**Table 5:** Descriptive statistics of the index values

WQI						
Date	N_WQI_	Mean	Min.	Max.	Q 25	Q 75
2013	40	147.31	52.78	306.73	91.81	180.32
2017	40	78.54	25.24	282.55	44.30	91.84
2018	40	102.92	30.00	403.59	60.98	113.69
2019	40	86.72	22.60	367.91	44.51	107.48
2021	37	72.48	18.89	220.84	39.74	97.67
2022	34	72.23	18.27	243.41	46.49	79.75
Cd						
Date	N_Cd_	Mean	Min.	Max.	Q 25	Q 75
2013	40	6.41	0.07	18.40	2.20	10.08
2017	40	4.75	0.00	26.40	1.30	6.68
2018	40	4.34	0.00	15.06	1.30	6.72
2019	40	5.38	0.00	21.31	1.51	8.45
2021	37	4.62	0.00	26.03	0.90	5.86
2022	34	6.11	0.09	26.32	1.31	8.90

**Fig. 4:**
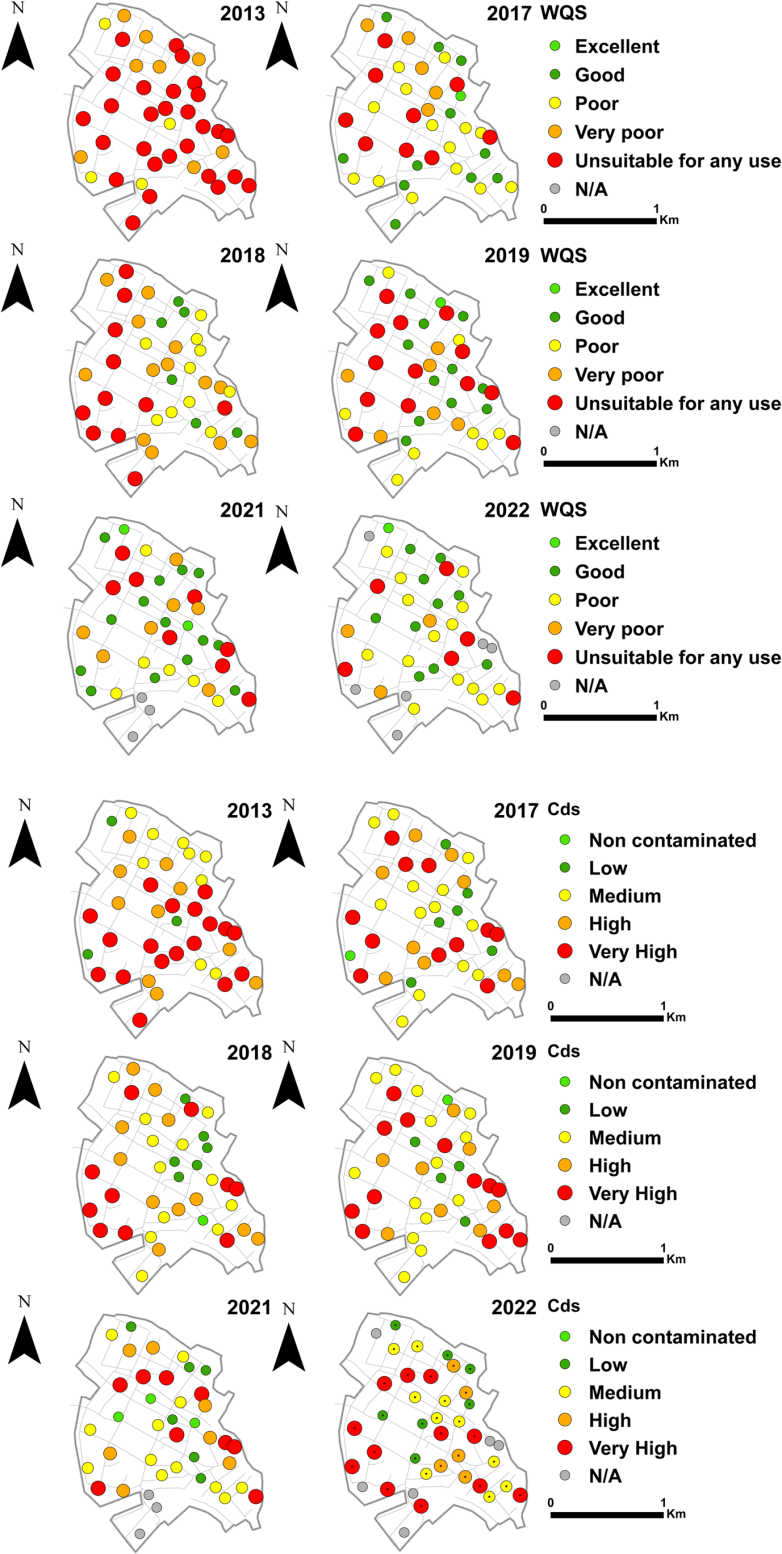
Water quality indices in the years before (2013) and after (2017, 2018, 2019, 2021, 2022) the construction of the sewerage network

**Fig. 5:**
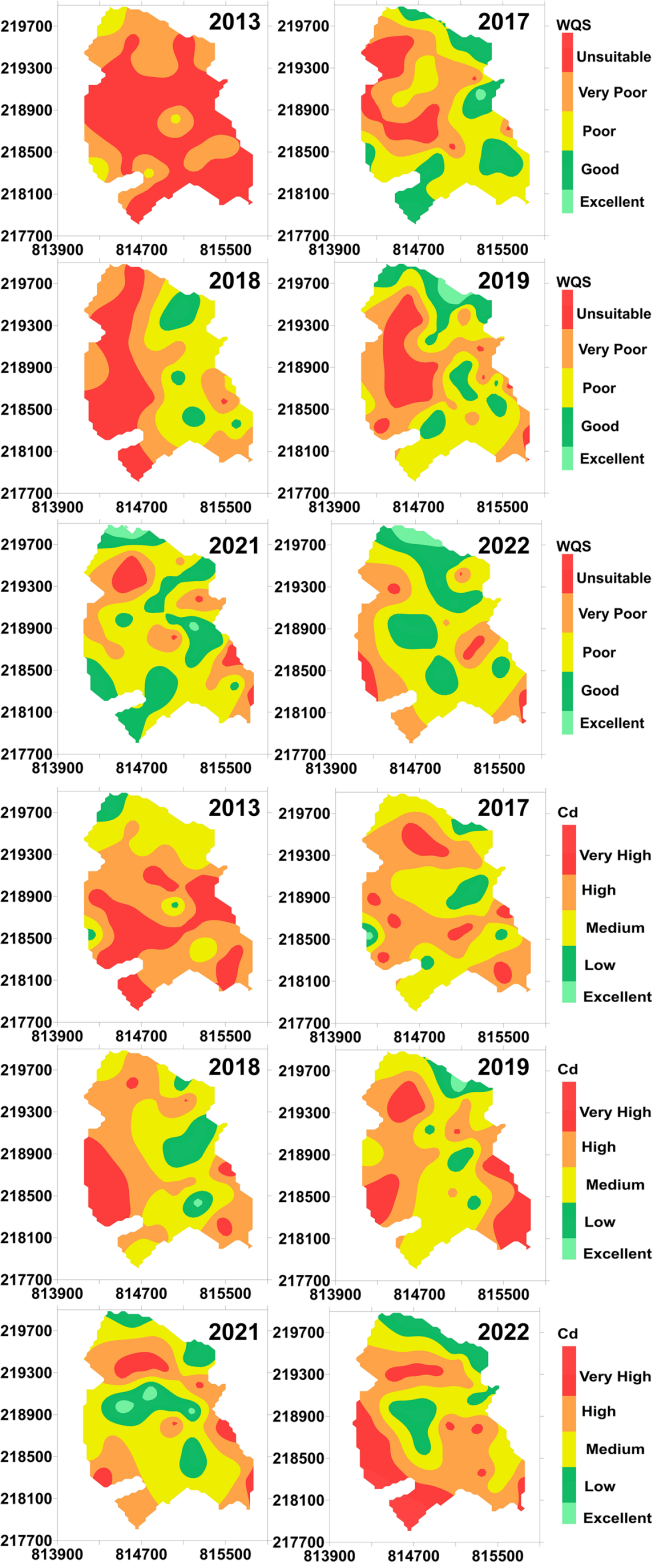
Spatial distribution of water quality indices in the years before (2013) and after sewerage (2017, 2018, 2019, 2021, 2022)

In order to reveal the spatial and temporal evolution of the water quality status of the monitoring wells and the degree of contamination, each index was plotted on thematic point maps for each sampling year (Fig. 4). Since a large number of samples were available, interpolated time-series distribution maps of water quality status and pollution levels were also made (Fig. 5).

In the year prior to construction of the sewerage network (2013), both indices showed contamination in a significant part of the settlement, while the northern areas of the settlement were moderately contaminated. The differences between WQS and Cd were most noticeable in the extent of heavily polluted–contaminated sites.

In the 3rd year after the construction of the sewerage network (2017), the proportion of heavily contaminated sites decreased significantly. In the inner areas of the settlement, the degree of pollution and the state of water quality decreased from ranks 4–5 to ranks 3–4. For both indices, the “Good” water quality status can be detected in the northern and central parts of the settlement—in the case of WQS, also in the southern areas—at varying magnitudes. The extent of these parts of the settlement is significantly greater in the case of WQS than in the case of Cd. This difference from WQS is more expressed in the southern part of the settlement. Both in terms of water quality status and degree of contamination, a similar pattern emerges in all studied years.

In the 4th year after the construction of the sewerage network (2018), the western part of the settlement can be considered again heavily polluted, but at the same time, the central and eastern parts showed significantly lower levels of pollution (medium and good) in extent compared to 2013. In 2019, there is an improvement in water quality compared to 2018, which is typically seen in the western areas of the settlement. The extent of the low-pollution running across the settlement from north to south increased for both indices.

In the 7th year after the construction of the sewerage network, the contaminated areas decreased significantly, at the same time the improved water quality became more pronounced from the central and N- NE parts of the settlement. The extent of the formerly typical N–S oriented high pollution zone running across the entire area of the settlement, has shrunk further.

In the 8th year after the construction of the sewerage network (2022), the state of water quality and the degree of pollution deteriorated compared to the previous year. For both indices, the extent and spatial configuration of low-pollution areas in the settlement decreased. The “Good” water quality status in the northern and central areas of the settlement can be still detected in varying degrees. However, the extent of these parts of the settlement is more homogeneous in the case of WQS than in the case of Cd.

### Cloud-based public access in term of geovisualization

**Fig. 6:**
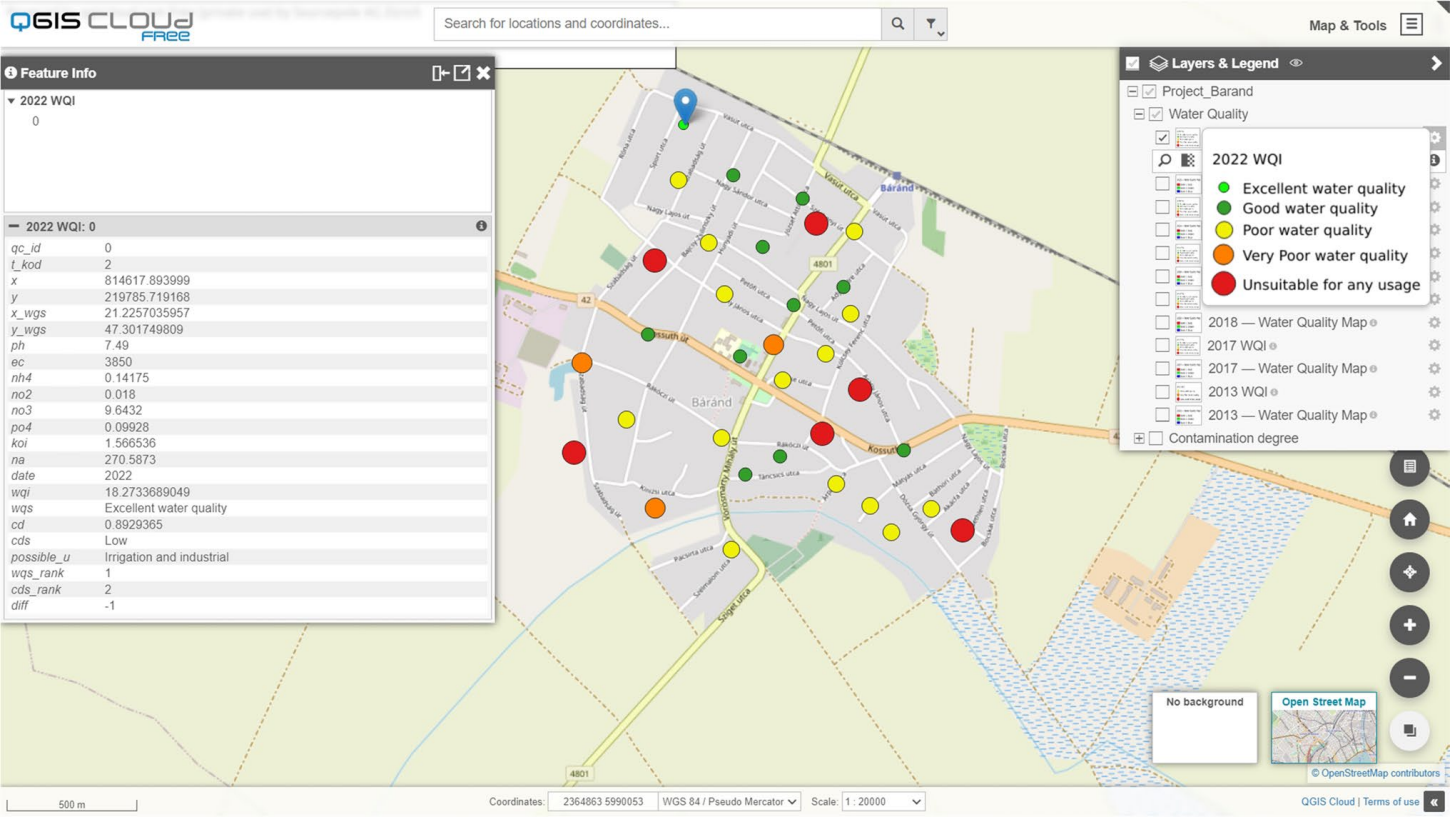
Results of geovisualization in QGIS Cloud platform

Following the geoinformatic processing and database creation of the water quality data the time series geodatabase was made freely available using the QGIS Cloud module (https://qgiscloud.com/Barand/Project_Barand). The geovisualized vector and raster layers present water quality index and contamination degree index categories with graduated symbols. The published cloud-based Web map shows the water quality and degree of contamination databases in the investigated years which can be zoomed in and out. We can use the Search by location function to the desired area in the Open Street Map base map, thereby informing the user about the spatial and temporal distribution of the water quality status and degree of contamination. In addition to navigating on the web map, we can control the visibility of each vector and raster layers. Metadata and descriptive information about the monitoring wells can be queried, and results geovisualizing from the databases are visible to users (Fig. 6). In the context of geovisualization, a cloud-based web map of a project is typically created not only for the community, but for individual use. As a result, the primary task of visualized spatial data is to provide data on water quality and to describe the purification processes following the construction of the sewerage network. In this sense, the spatial display of water quality data and communication with the user are complementary events in cloud-based map use.

### Comparative analysis of WQI and Cd

Following the categorization of the water quality status and contamination degree of the water samples, the difference between the ranking of the same water sample using the two indices was studied. In the course of the analysis the data over the 6 years were not separated they were used as one dataset.

Correlational tests were made in order to determine the strength of the correlation between the water quality indices (Table 6). The results revealed that there is a significant connection (*p* > 0.01) between both indices. Correlation between WQS and Cd shows moderate strength for the connection (*r* = 0.557).

**Table 6:** Correlation matrix of water quality indices based on the data of 6 years

Correlation coefficient	WQS	C_d_
WQS	1	
C_d_	0.557	1

Significance level of correlation: 0.01

The difference between the ranks based on the water quality indices was maximum ± 2 on a scale of five in 97.4% of the cases. A difference of occurred only 6 times out of the 231 cases. The difference between WQS and Cd was − 1.0+1 in 82.7% of the cases (Table 7).

**Table 7:** Differences between categories for 6 years

Difference between rank	WQS-C_d_	
	Number of monitoring wells	%
− 3	6	2.6
− 2	23	10
− 1	39	16.9
0	94	40.7
1	58	25.1
2	11	4.8
Total	231	100

**Fig. 7:**
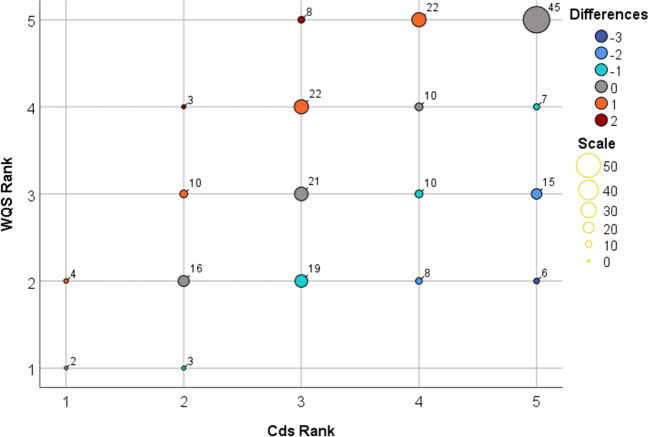
Scatter plot of WQS vs. Cds

The number of monitoring wells in the same category were examined and the differences between the established ranks for the given category (Fig. 7). In the rank 1 category, representing the best water quality, 5 and 6 monitoring wells were classified based on WQS and Cd, respectively; the category of 2 wells were matching. The most diverse differences were found in rank 2, since all 5 categories, although to varying degrees and differences, appear. There were 53 monitoring wells in the rank 2 category based on WQS and 32 monitoring wells based on Cd, 16 of which were ranked in the same category. The rank 3 category, which indicates moderate contamination, has the second highest number of classifications without category changes (*N* = 21). The second smallest number of matches is shown by wells in the rank 4 category, where, in addition to the 42 monitoring wells of the WQS, 10 of the 50 monitoring wells of Cd were classified in the same category. The highest number of matches was found in rank 5, which represents the most contaminated category, as 45 monitoring wells were classified that were heavily contaminated based on both WQS and Cd.

### Analysis of the impact of sewerage network construction on water quality indices

We also aimed to support the connection between changes in water quality indicators and the sewerage network with geostatistical analyses. In order to determine the changes in water quality after the construction of the sewerage network and the differences in the contamination of the monitoring wells, the data series for the 6 years was divided into pre-sewerage (2013) and post-sewerage (2017–2022) categories.

**Fig. 8:**
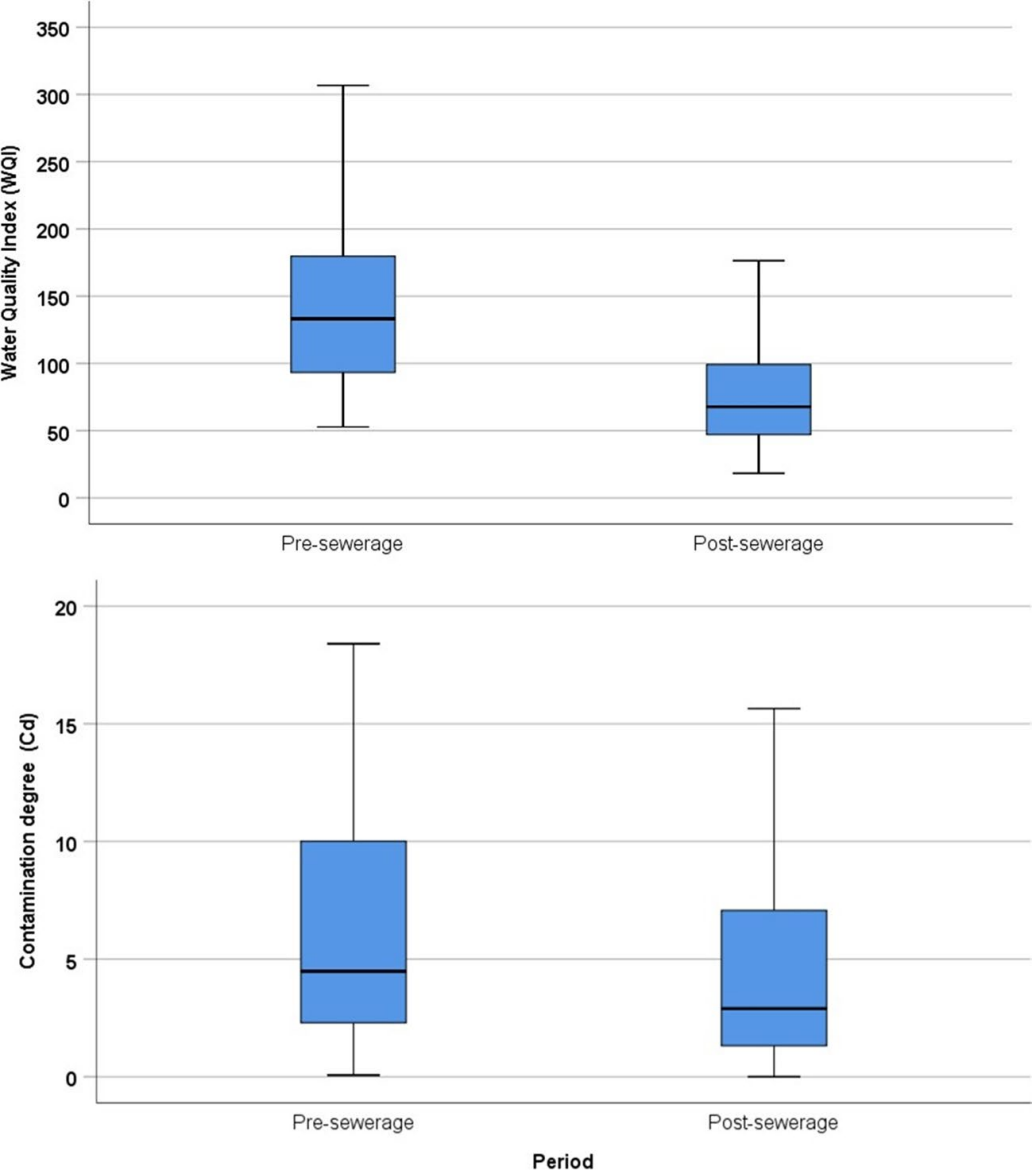
Index values in the years before and after the establishment of the sewerage network

Using Wilcoxon’s signed rank test significant differences were revealed between the pre- and post-sewerage data series for the indices, which is explained by the impacts of the sewerage network (Table 8, Fig. 8). The strongest significance (*p* = 0.0000) was found in the case of the WQI index values, where the *Z* value was − 4.463. For Cd index values, nearly identical values were obtained, which significantly indicate the positive influences of sewerage albeit to a lesser extent (Table 8).

**Table 8:** Results of Wilcoxon signed rank test

	WQI_pre-sewerage_-WQI_post-sewerage_	Cd_pre-sewerage_-Cd_post-sewerage_
*Z*	− 4.463	− 2.554
Asymp. sig. (2-tailed)	0.000	0.11

To determine whether the results of the pre-sewerage and post-sewerage periods can be separated from each other, a discriminant analysis was performed. The Wilks-Lambda test showed a significant (*p* = 0.000) value. 80.5% of the cross-checked values were successfully classified in the appropriate group (Table 9). However, the correct classification of 81.4% of the samples also indicates a significant difference between the pre-sewerage and post-sewerage periods. Based on this, the construction of the sewerage network has led to positive changes in the quality of the monitoring wells. The results of the statistical analyses confirmed that after the construction of the sewerage network the water quality index values of the studied monitoring wells improved due to a significant reduction in wastewater discharge, which was confirmed by research carried out in other urban environments (Fylypchuk et al. 2017; Mester et al. 2017; Bugajski et al. 2019; Schuler et al. 2019; Ahmad and Ghanem 2021).

**Table 9:** Results of discriminant analysis before and after the construction of the sewerage network

Classification results^a,c^					
		Group	Predicted group membership		Total
			1	2	
Original	Count	Pre-sewerage	25	15	40
		Post-sewerage	28	163	191
	%	Pre-sewerage	62.5	37.5	100
		Post-sewerage	14.7	85.3	100
Cross-validated^b^	Count	Pre-sewerage	24	16	40
		Post-sewerage	29	162	191
	%	Pre-sewerage	60.0	40.0	100
		Post-sewerage	15.2	84.8	100

^a^81.4% of original grouped cases correctly classified

^b^Cross-validation is done only for those cases in the analysis. In cross-validation, each case is classified by the functions derived from all cases other than that case

^c^About 80.5% of cross-validated grouped cases correctly classified

## Limitation of study

The most important task of qualitative assessment of water quality based on monitoring data is to interpret information on the state of water quality (Ball and Church 1980; Backman et al. 1998; Bouslah et al. 2017). Thus, water quality indices are tools that greatly minimize the amount of data and simplify the expression of water quality status. However, they are unsuitable for a comprehensive analysis of the relationship between the physicochemical-biological parameters used for this purpose, and for determining the relationships between the parameters shaping water quality or their natural or anthropogenic origin (Tirkey et al. 2013). According to Uddin et al. (2021), water quality indices generally share similar structures, but the specific details of the four main components (parameters, sub-indices, weightings, and aggregation) can vary significantly (Uddin et al. 2021). Their study also highlighted issues related to water quality indices development, such as eclipsing and uncertainty (Juwana et al. 2016; Sutadian et al. 2018). Although many indices are designed to be easily transferable to different sites, their applications are often specific to the study area. The number and types of parameters included in WQI, the weightings assigned to particular parameters, and the criteria (limit values) used to develop sub-index values can vary greatly (Swamee and Tyagi 2007; Abbasi and Abbasi 2012). Therefore, there is little uniformity among models, making it difficult to compare applications across different study areas (Uddin et al. 2021).

Our study shows a significant positive change in the groundwater quality of the water reservoir of the settlement evaluated on the basis of water quality indicators. A comparative analysis of the applied indices highlights the differences between them, which is also verified by case studies conducted in other settlements (Franz et al. 2022; Pham et al. 2022). The WQI index is more sensitive to changes in certain parameters (e.g., PO_4_^3−^, NH_4_^+^) than the Cd index due to the different weighting of the input data (Mester et al. 2020; Guasmi et al. 2022). The Cd index does not take into account values below the limit, even if they are very close to it (Backman et al. 1998). Due to this, when choosing water quality indices, it is necessary to take into account not only its field of application (agricultural, industrial, urban environment), but also the expected important hydrochemical–water physical parameters for calculating index values.

The Web geovisualization of the water quality data produced during the research was realized using several geoinformatics tools and systems. However, in relation to the geoinformatic processing of raw measurement data, the paradigm shift in traditional GIS-based systems has to be mentioned (Agrawal and Gupta 2017). Cloud-based WebGIS systems are now capable not only of managing geodatabases but also of performing complex tasks (e.g., defining data structure, quality control and validation of input data, Web data visualization) in real time by integrating task-oriented modules. One of the most important benefits of this is that there is no need for the post-processing of data on an additional platform or software environment. Research on WebGIS architectures agrees that in addition to cloud-based technologies, the presence of artificial intelligence and neural nets will be unavoidable in the long run-in innovative developments based on spatial data (Kholoshyn et al. 2019; Lavallin and Downs 2021; Nader et al. 2022).

## Conclusion

Our study examined the changes in groundwater quality after the construction of a sewerage network and the elimination of wastewater outflow in a municipal environment, using water quality indices and various geospatial and statistical analyses. The geodatabase obtained on the basis of long-term monitoring between 2013 and 2022 was made available online in WebGIS cloud-based systems in order to make environmental data as widely accessible as possible. The results for the pre-sewerage period highlight the strong contamination of the groundwater resources of the settlement, due to, among other things, improper storage of domestic wastewater and unresolved wastewater treatment until 2014. The applied water quality indices classified the majority of the groundwater wells studied into the contaminated or heavily contaminated categories. During the monitoring period following the construction of the sewerage network (2017–2022), clear purification of the groundwater was found, which is the result of the elimination of local point-like pollution sources. During this period, increasing areas of the settlement showed adequate or good water quality values. The marked positive changes were confirmed with Wilcoxon tests and discriminant analyses. In the discriminant analyses, it was possible to determine with more than 80% probability, whether the given water sample originated in the year before or after the construction of the sewerage network, based on water chemical parameters. However, the level of contamination of the groundwater is still detectable, and values exceeding the limits are encountered in the case of all of the studied parameters. This clearly shows that organic and inorganic substances accumulated in the area are still present. Further, long-term monitoring is necessary to understand the dynamics of the purification processes. Making these available for the society on a Web-based platform may contribute to appropriate environmental measures and strategies.

## Data Availability

The datasets generated during and/or analyzed during the current study are available from the corresponding author on reasonable request.
